# Coping with cancer genes altered by copy number

**DOI:** 10.18632/oncotarget.6215

**Published:** 2015-10-21

**Authors:** Natalie K. Wolf, David A. Largaespada, Branden S. Moriarity

**Affiliations:** Department of Pediatrics, Masonic Cancer Center, University of Minnesota, Twin Cities, MN, USA

**Keywords:** Chromosome Section, functional genomics, TCGA, transposons, mouse

Cancer is a genetically heterogeneous disease in which many, many genes or gene products have altered expression or activity due to a variety of genetic and epigenetic mechanisms. These alterations result in activation of oncogenic processes that do not occur in normal somatic cell counterparts. With advances in high-throughput sequencing and other technologies over the past decade, genomic analysis of various cancers has led to new insights of these oncogenic processes. It was recently discovered in a meta-analysis of many human tumor types, that they are characterized as having either a large number of somatic mutations (M class) or copy number of alterations (C class), but usually not both [1]. Many common and lethal human cancers, such as glioblastoma multiforme and acute myeloid leukemia, have been identified as M class tumors. Whole genome sequencing technologies (WGS) have identified many of the genes that drive these cancers when mutated, some of which are good targets for therapy. However, C class tumors present a challenge for the identification of drivers that would make good targets for therapy. This is because the gene copy number alterations often affect large regions of the genome, containing many genes, in any one case. Moreover, we know from recent chromosome engineering studies in mice, and other studies, that even low-level gene copy number alterations can contribute to the formation of cancer [2]. Being able to determine elusive C class driver genes could aid in our understanding of the oncogenic processes in these misunderstood tumors, and suggest new ways to treat these forms of cancer.

*Sleeping Beauty* (SB) transposon-based forward genetic screens in mice have been successfully used to identify driver genes in both C and M class types of cancer, but is particularly informative in C class tumors where genomic analysis of human tumors is less informative [3, 4]. Analysis of recurrent transposon insertion sites reveals common insertion site (CIS)-associated genes, many of which are specific to the cancer being studied. Using CIS-associated genes as an enriched list of candidate driver genes combined with array comparative genome hybridization (aCGH) or WGS of human C class tumors has allowed for the validation of CISs corresponding to human genes with meaningful gains or losses in copy number [3, 4]. Thus, the *SB* mutagenesis system provides the capability to identify C class tumor driver genes via cross species genomics comparisons.

In our recent work, we performed a forward genetic screen using the conditional *SB* mutagenesis system to identify driver genes involved in development and metastasis of osteosarcoma, a prototypical C class tumor type [4]. Osteosarcoma is the most common primary bone cancer and the third most common cancer in children and adolescents. Osteosarcomas are among the most disordered cancers in terms of whole-chromosome and copy number variations. Further, as is common to C class tumors, copy number variations in osteosarcomas typically affects large regions of the genome making it almost impossible to identify specific drivers whose low-level amplification or loss leads to tumor development. By utilizing an *SB* forward genetic screen, we were able to identify hundreds of significant CIS-associated genes for osteosarcoma, including both previously noted (*MYC*, *CAPRIN1*, and *AKT2*) and novel candidate drivers (*SEMA4D*, *SEMA6D*, and *ZNF217*). Interestingly, several candidate oncogenes involved in axon guidance were identified, including *SEMA4D* and *SEMA6D,* which we functionally validated as oncogenes in human osteosarcoma [4].

Paralleling *SB* screens are other functional genomics approaches, such as lentiviral and retroviral focused libraries that can be used to identify C class drivers. Focused shRNA libraries have been used to downregulate tumor suppressor genes in mice to identify important tumor suppressor genes in the development of cancers, such as hepatocellular carcinomas [5]. Focused libraries of cDNAs, and shRNAs, have been used to define copy number altered oncogenic drivers and tumor suppressors of ependymoma [6]. These experiments utilized focused libraries based on the genes in regions that are recurrently gained or lost in sets of human tumors that have been analyzed. Recently, another approach that could be utilized to identify C class drivers has emerged, namely genome-wide CRISPR/Cas9 based inactivation screens [7].

C class drivers have been successfully identified in a variety of ways including *SB* screens, lenti/retroviral focused cDNA or shRNA libraries, and now potentially through the use of the CRISPR/Cas9 system. These drivers are likely to reveal druggable targets, and provide hope for new clinical trials in these forms of cancer. One such example is the potential use of the humanized antibody XV15 for targeting SEMA4D, a novel driver of osteosarcoma found through our *SB* screen [4]. Identification of these novel C class drivers will greatly improve our knowledge of new oncogenic processes and potential therapeutic targets.
